# Expression of Polyamine Oxidase in Fibroblasts Induces MMP-1 and Decreases the Integrity of Extracellular Matrix

**DOI:** 10.3390/ijms231810487

**Published:** 2022-09-10

**Authors:** Hae Dong Jeong, Jin Hyung Kim, Go Eun Kwon, Seung-Taek Lee

**Affiliations:** 1 Department of Biochemistry, College of Life Science and Biotechnology, Yonsei University, Seoul 03722, Korea; 2 Molecular Recognition Research Center, Korea Institute of Science and Technology, Seoul 02792, Korea

**Keywords:** polyamine oxidase, fibroblast, reactive oxygen species, polyamine, extracellular matrix, matrix metalloproteinase-1

## Abstract

Polyamine oxidase (PAOX) (*N*^1^-acetylpolyamine oxidase) is a major enzyme in the polyamine catabolism pathway that generates hydrogen peroxide. Hydrogen peroxide plays a crucial role in skin aging via extracellular matrix (ECM) degradation by increasing the matrix metalloproteinase-1 (MMP-1) levels. We analyzed the integrity of the ECM in foreskin fibroblasts using PAOX expression. PAOX increased the MMP-1 secretion and type Ι collagen degradation in 2D and 3D cultures of fibroblasts, respectively. Similarly, PAOX overexpression increased the messenger ribonucleic acid (mRNA) level of *MMP-1*. PAOX expression induced polyamine catabolism, decreased the spermine levels, and increased the putrescine levels. However, the exogenous polyamine treatment did not change the MMP-1 and type I collagen levels as much as PAOX expression. PAOX expression increased the reactive oxygen species (ROS) production in fibroblasts, and exogenous hydrogen peroxide increased both the ROS production and MMP-1 secretion. Furthermore, *N*-acetylcysteine, an antioxidant, reversed the PAOX-induced ROS production and MMP-1 secretion. PAOX induced the signaling pathways that activate activator protein-1 (AP-1) and nuclear factor kappa-light-chain-enhancer of activated B cells (NF-κB), which are important transcription factors for *MMP-1* transactivation. We concluded that PAOX increased the ROS levels in fibroblasts, leading to an increase in MMP-1 expression. Therefore, we propose that PAOX is a potential target molecule in protecting the ECM integrity.

## 1. Introduction

Polyamines, including spermidine (SPD), spermine (SPM), and the precursor diamine putrescine (PUT), are polycationic alkylamines. The intracellular levels of polyamines are controlled by several processes, such as anabolism, catabolism, absorption, and excretion. Polyamine anabolism, catalyzed by enzymes such as spermidine synthase (SRS) and spermine synthase (SMS), results in the production of SPD and SPM. The catabolism of polyamines produces PUT from SPD and SPM via polyamine oxidase (PAOX), spermidine/spermine *N*^1^-acetyltransferase (SSAT), and spermine oxidase (SMOX).

Polyamines are necessary for the growth and survival of mammalian cells [1]. The cationic properties of polyamines provide stability to nucleic acids and contribute to essential cellular processes, including deoxyribonucleic acid (DNA) replication, transcription, and translation [1,2]. The antioxidant activity of polyamines protects DNA and phospholipids from oxidative damage, and together with the membrane-stabilizing effect, polyamines contribute to anti-inflammatory activity [3,4,5,6]. However, excess polyamines are cytotoxic to mammalian cells [7] and are associated with pathological implications, such as cancer, stroke, and renal dysfunction [1].

PAOX is an oxidase that is involved in polyamine catabolism, along with SSAT. It produces SPD and PUT using *N*-acetyl SPM and *N*-acetyl SPD as substrates, respectively, which are generated from SPM and SPD by SSAT, respectively. During this reaction, catalyzed by PAOX, hydrogen peroxide (H_2_O_2_) is produced as a by-product of oxidation. Polyamine catabolism is enhanced in diverse pathological conditions, such as inflammation, traumatic brain injury, and acute kidney injury [1,4,8,9].

H_2_O_2_, a reactive oxygen species (ROS), is cytotoxic to various cells. It is produced in some organelles, such as peroxisomes by peroxisomal oxidases, mitochondria by electron transport chain reaction, and chloroplasts by the Mehler reaction [10]. Low concentrations of H_2_O_2_ are beneficial to cells as they induce cell proliferation, differentiation, and migration [11]. However, at high concentrations, beyond the endurance of cells, H_2_O_2_ damages DNA, RNA, and lipids [11,12], and promotes inflammation, tumor growth, metastasis, and apoptosis. Therefore, the human body employs several mechanisms to regulate the H_2_O_2_ levels using redox-controlling proteins, such as peroxiredoxins and glutathione peroxidases [11].

Matrix metalloproteinases (MMPs) are a family of zinc-dependent endopeptidases that are capable of cleaving most extracellular matrix (ECM) proteins. The MMP family consists of structurally and functionally related subfamilies, including collagenases, gelatinases, stromelysins, and membrane-type MMPs. MMP-1 is secreted by dermal fibroblasts, keratinocytes, and macrophages in the skin, and it plays an important role in the degradation of fibrillar collagens, such as type I and type III collagens in the skin ECM [13,14]. Fibrillar collagen degradation reduces the ECM integrity of the skin and forms wrinkles, which are an evident phenotype in skin aging. In fact, MMP-1 is induced by various conditions, such as inflammation and high ROS levels, which increase with aging [15,16]. Consistently, the messenger ribonucleic acid (mRNA) and protein levels of MMP-1 are higher in aged human dermis than those in young dermis [17]. Therefore, MMP-1 is a crucial marker of skin aging.

Inflammation is associated with an increase in polyamine catabolism, thereby decreasing the polyamine levels. In addition, skin aging that involves ECM degradation is influenced by ROS and inflammation. Here, we have investigated the role of PAOX, which reduces polyamines and generates H_2_O_2_, in modulating the ECM integrity in fibroblasts. In the present study, we analyzed the MMP-1 expression and type Ι collagen degradation in 2D and 3D cultures of fibroblasts, with or without PAOX expression. In addition, the effects of PAOX expression on the polyamine distribution, and the exogenous polyamine treatment on the MMP-1 secretion, were analyzed in the fibroblasts. We examined the ROS production using the PAOX expression level, and the ROS production and MMP-1 secretion using the exogenous H_2_O_2_ levels in the fibroblasts. Furthermore, the effects of an antioxidant on PAOX-induced ROS production and MMP-1 secretion were analyzed. Finally, we investigated the signaling pathways that are important for PAOX-induced MMP-1 induction in fibroblasts. Based on the results, we propose that PAOX expression plays a role in the ECM integrity in fibroblasts, and in the underlying molecular mechanisms of MMP-1 induction.

## 2. Results

### 2.1. PAOX Expression Increases MMP-1 Secretion in Fibroblasts

**Figure 1:**
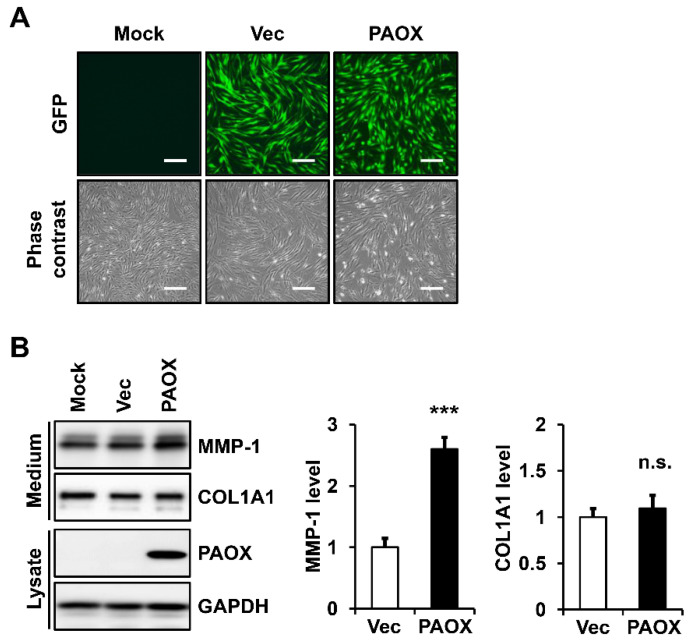
Effect of polyamine oxidase (PAOX) expression on the secretion of matrix metalloproteinase-1 (MMP-1) and type I collagen in fibroblasts. (**A**) Fibroblasts were mock-infected or infected with a vector control or PAOX viruses. The fibroblasts were analyzed using fluorescence and phase contrast microscopy. Magnification: ×100. Bar = 200 µm. (**B**) Fibroblasts were incubated in a serum-free and phenol-red-free Dulbecco’s modified Eagle’s medium (DMEM) for 24 h. Conditioned media and cell lysates were analyzed using Western blotting with anti-MMP-1, anti-pN-COL1A1, anti-PAOX, and anti-glyceraldehyde-3-phosphate dehydrogenase (GAPDH) antibodies. Protein levels were measured using ImageJ software and were normalized to GAPDH levels. In graphs, each value represents the mean ± standard deviation of three independent experiments. *** *p* < 0.001 vs. vector control. n.s.: not significant.

To examine the effect of the ectopic expression of PAOX on the ECM modulation in fibroblasts, we analyzed the secretion of MMP-1 and type I collagen using Western blot analysis. The virus-encoded green-fluorescent-protein (GFP) expression levels were similar in virus-infected fibroblasts expressing PAOX and the vector control at 24 h after infection, suggesting that the titers of the viruses in both cells were similar (Figure 1A). The analysis of the fibroblasts revealed that the PAOX expression significantly increased the MMP-1 secretion compared with that of the infection with the control virus and mock infection, but it did not significantly affect the type I collagen secretion (Figure 1B).

### 2.2. PAOX Expression Increases Type I Collagen Degradation in 3D Culture of Fibroblasts

**Figure 2:**
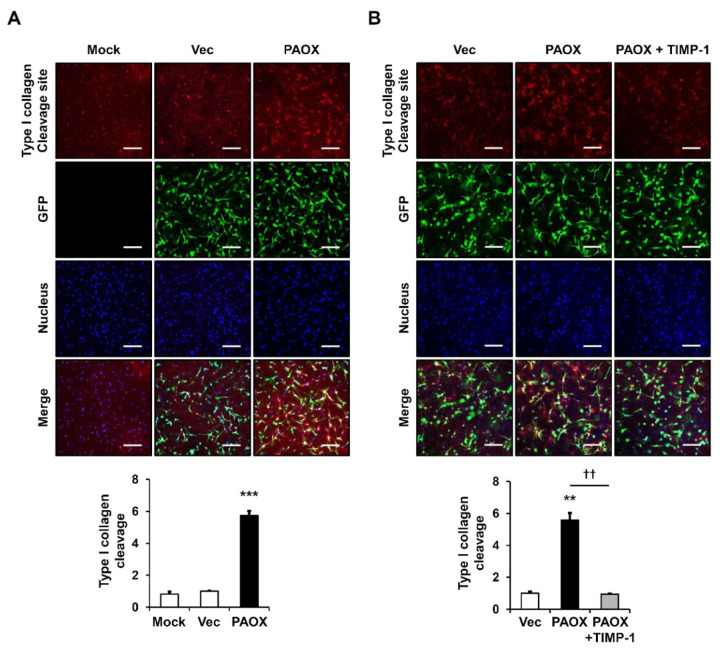
Effect of PAOX expression on type I collagen degradation in a 3D culture of fibroblasts. Fibroblasts mock-infected or infected with vector viruses or PAOX viruses were embedded in a type I collagen matrix and incubated in a serum-free and phenol-red-free DMEM for 24 h (**A**) without or (**B**) with 1 μg/mL tissue inhibitor of metalloproteinases-1 (TIMP-1). Fibroblasts were subjected to immunofluorescence (IF) staining for type I collagen degradation using a rabbit anti-type I collagen cleavage-site antibody and a Rhodamine Red X-conjugated anti-rabbit IgG antibody. Nuclei were stained with Hoechst 33258 and were used for normalization of fluorescence values. The fluorescence levels of type I collagen degradation were measured using ImageJ software and are presented as a graph. Each value represents the mean ± standard deviation of three independent experiments. ** *p* < 0.01 and *** *p* < 0.001 vs. vector control. †† *p* < 0.01 vs. PAOX. Magnification, ×100. Bar = 200 µm.

We then examined the degradation of type I collagen in the collagen-embedded 3D cultures of foreskin fibroblasts, with or without PAOX overexpression. To examine the cleavage of type I collagen by MMP-1, immunofluorescence (IF) staining of the 3D gel was performed with a type I collagen cleavage-site antibody that recognizes three-fourths of the type I collagen fragment. The virus-encoded GFP levels were similar in the vector control and PAOX-expressing cells, indicating that the virus titers were similar in both cells. Under these 3D-culture conditions, the PAOX expression significantly increased the type I collagen degradation compared with that of the mock-infected and vector control cells (Figure 2A). The treatment of the tissue inhibitor of metalloproteinases-1 (TIMP-1), which specifically inhibit MMPs, including MMP-1 [18], significantly reduced the type I collagen degradation that was enhanced by PAOX expression (Figure 2B).

### 2.3. PAOX Expression Increases MMP-1 mRNA Level in Fibroblasts

**Figure 3:**
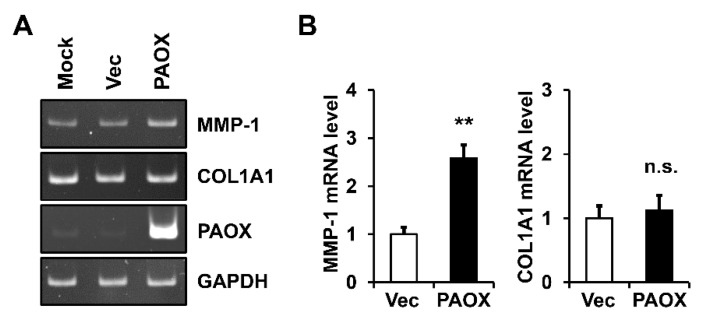
Effect of PAOX expression on *MMP-1* messenger ribonucleic acid (mRNA) levels in fibroblasts. Fibroblasts mock-infected or infected with vector viruses or PAOX viruses were incubated in a serum-free DMEM for 12 h. Levels of *MMP-1*, *COL1A1*, *PAOX*, and *GAPDH* mRNAs were assessed using (**A**) conventional and (**B**) real-time reverse transcription polymerase chain reaction (RT-PCR) analyses. Graphs depict the relative levels of *MMP-1* and *COL1A1* mRNAs versus vector-infected cells, obtained using real-time RT-PCR analysis. Each value represents the mean ± standard deviation of three independent experiments. ** *p* < 0.01 vs. vector control. n.s.: not significant.

To determine whether the PAOX-mediated increase in the MMP-1 protein levels was due to an increase in its mRNA level, the levels of *MMP-1* mRNA in the fibroblasts, with or without PAOX expression, were determined using conventional and real-time reverse transcription polymerase chain reaction (RT-PCR). PAOX expression significantly upregulated the *MMP-1* mRNA levels, unlike what we observed in the mock-infected and control vector cells. However, PAOX did not significantly change the collagen type I alpha 1 chain (*COL1A1*) mRNA levels in the same samples (Figure 3A,B).

### 2.4. PAOX Expression Activates Polyamine Catabolism in Fibroblasts

**Figure 4:**
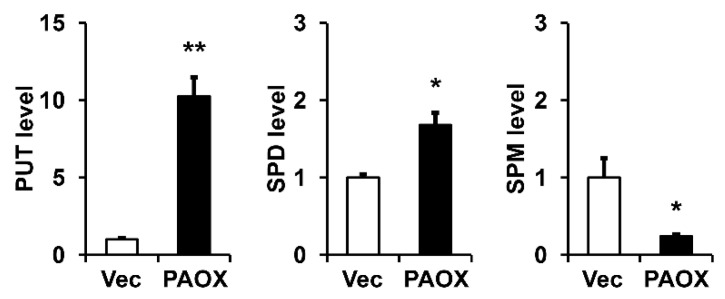
Effect of PAOX expression on polyamine distribution in fibroblasts. Fibroblasts infected with vector viruses or PAOX viruses were incubated in a serum-free DMEM for 24 h, and the levels of polyamines in the cell lysates were analyzed using gas chromatography–mass spectrometry. Graphs depict putrescine (PUT), spermidine (SPD), and spermine (SPM) levels relative to vector-infected cells. Each value represents the mean ± standard deviation of three independent experiments. * *p* < 0.05 and ** *p* < 0.01 vs. PUT, SPD, or SPM levels in vector control.

Because PAOX is involved in the catabolism of polyamines, we compared the intracellular distribution of PUT, SPD, and SPM in the fibroblasts, with or without PAOX expression, measured by gas chromatography–mass spectrometry (GC-MS). The PAOX expression significantly decreased the SPM levels, moderately increased the SPD levels, and significantly increased the PUT levels, demonstrating polyamine catabolism (Figure 4).

**Figure 5:**
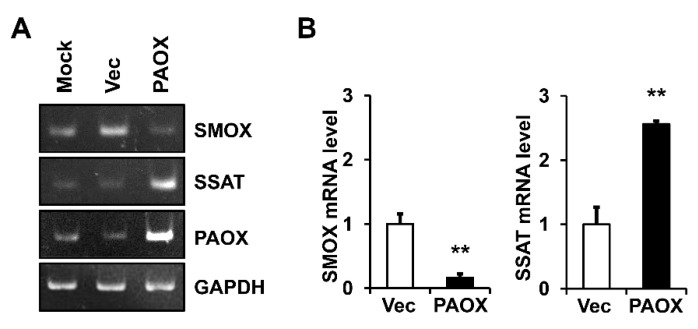
Effect of PAOX expression on spermine oxidase (*SMOX*) and spermidine/spermine *N^1^*-acetyltransferase (*SSAT*) mRNA levels in fibroblasts. Fibroblasts mock-infected or infected with vector viruses or PAOX viruses were incubated in a serum-free DMEM for 12 h. The levels of *SMOX*, *SSAT*, *PAOX*, and *GAPDH* mRNAs were assessed using (**A**) conventional and (**B**) real-time RT-PCR analyses. Graphs depict the relative levels of *SMOX* and *SSAT* mRNAs versus vector-infected cells using real-time RT-PCR analysis. Each value represents the mean ± standard deviation of three independent experiments. ** *p* < 0.01 vs. vector control.

We analyzed the mechanism by which PAOX expression alone enhanced the polyamine catabolism. In polyamine catabolism, SMOX alone catalyzes the oxidation of SPM to SPD, and two enzymes, SSAT and PAOX, catalyze the oxidation of both SPM to SPD and SPD to PUT [1]. To determine whether PAOX expression affects the expressions of SMOX and SSAT, the *SMOX* and *SSAT* mRNA levels in the fibroblasts, with or without PAOX expression, were determined using conventional and real-time RT-PCR. The PAOX expression significantly reduced the *SMOX* mRNA levels and increased the *SSAT* mRNA levels (Figure 5A,B). Therefore, we found that PAOX expression activated the SSAT/PAOX-mediated polyamine catabolic pathway while suppressing SMOX-mediated SPM catabolism.

### 2.5. Exogenous Polyamines Have Little Effect on MMP-1 Secretion in Fibroblasts

**Figure 6:**
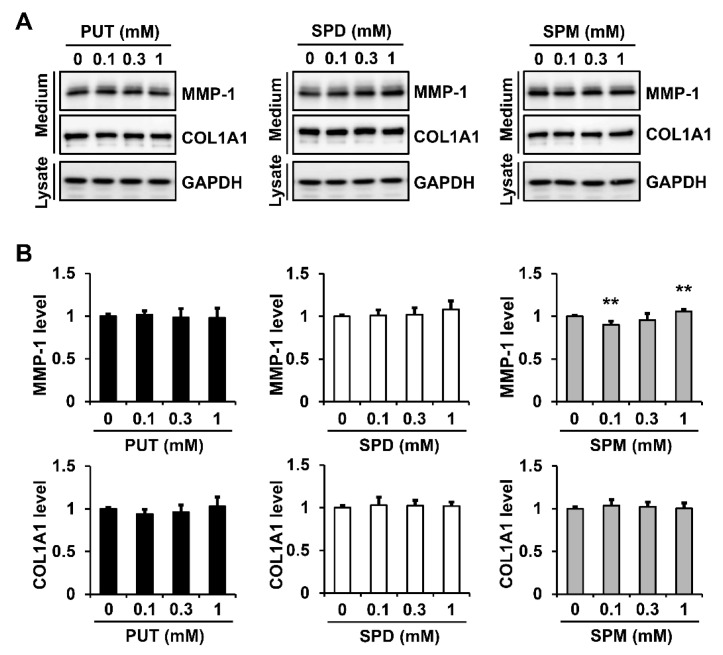
Effect of exogenous polyamines on the secretion of MMP-1 and type I collagen in fibroblasts. Fibroblasts were incubated in a serum-free DMEM with various concentrations of PUT, SPD, and SPM for 24 h. (**A**) Conditioned media and cell lysates were analyzed using Western blotting with anti-MMP-1, anti-pN-COL1A1, and anti-GAPDH antibodies. (**B**) Protein levels were measured using the ImageJ software and were normalized to GAPDH levels. Each value represents the mean ± standard deviation of five independent experiments. ** *p* < 0.01 vs. untreated cells.

To examine whether changes in the polyamine distribution affected the MMP-1 secretion, we analyzed the effect of exogenous polyamines (0.1, 0.3, and 1 mM) on the MMP-1 and type I collagen secretion. Treatment with PUT and SPD did not significantly affect the MMP-1 secretion. However, the SPM treatment caused some changes in the MMP-1 secretion: 0.1 mM SPM decreased the MMP-1 secretion to 90.1 ± 4.2%, compared with that of the untreated sample, and 1 mM SPM increased the MMP-1 secretion to 105.8 ± 2.2%. The polyamine treatment had no significant effect on the type I collagen secretion (Figure 6A,B). These results indicated that changes in the polyamine distribution had little effect on the MMP-1 secretion.

### 2.6. PAOX Expression Increases Intracellular ROS Levels in Fibroblasts

**Figure 7:**
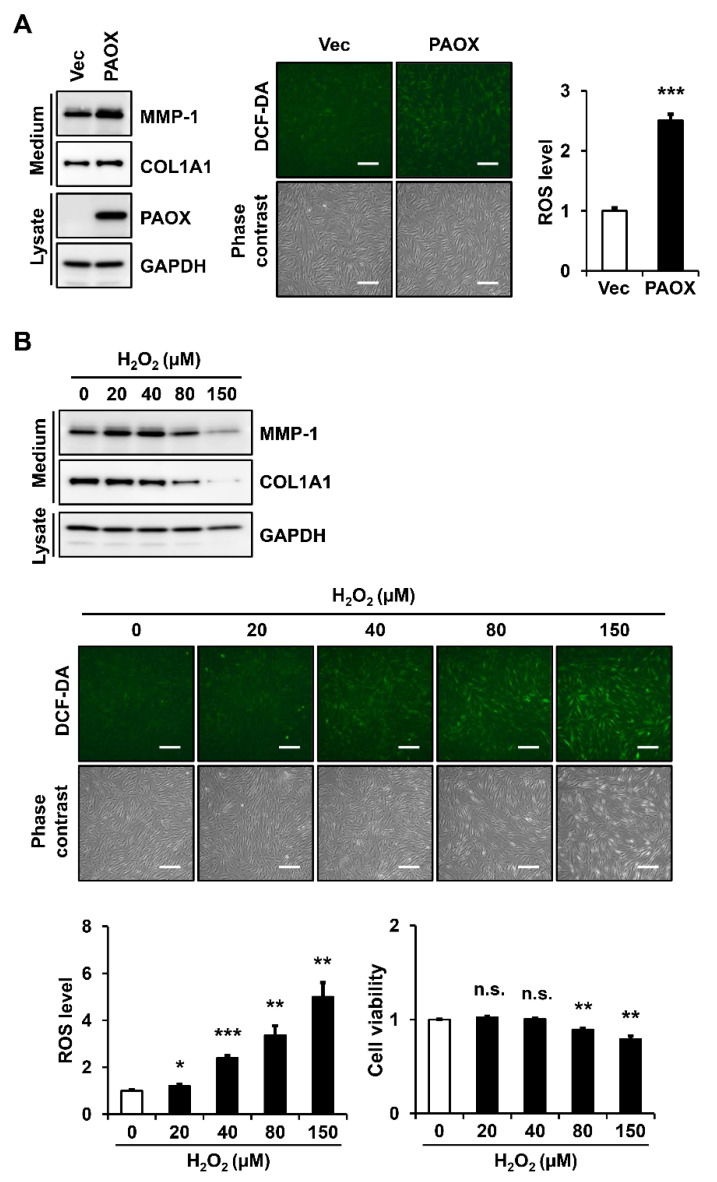
Effect of PAOX expression on reactive oxygen species (ROS) generation in fibroblasts. (**A**) Fibroblasts infected with GFP-deleted vector viruses or GFP-deleted PAOX viruses were incubated in a serum-free and phenol-red-free DMEM for 24 h. (**B**) Fibroblasts were incubated in a serum-free and phenol-red-free DMEM for 24 h in the presence of various concentrations of hydrogen peroxide (H_2_O_2_). Conditioned media and cell lysates were analyzed using Western blotting with anti-MMP-1, anti-pN-COL1A1, anti-PAOX, and anti-GAPDH antibodies. Intracellular ROS levels were detected by green fluorescence at 525 nm after staining with 10 μM DCF-DA for 30 min. ROS levels were assessed using the ImageJ software, normalized with cell numbers counted by crystal violet staining, and shown in graphs relative to the ROS levels in (**A**) vector-infected cells or (**B**) untreated cells. Cell viability was measured by the crystal violet staining and depicted in a graph relative to that in untreated cells. Each value represents the mean ± standard deviation of three independent experiments. * *p* < 0.05, ** *p* < 0.01, and *** *p* < 0.001 vs. control. n.s.: not significant. Magnification: ×100. Bar = 200 µm.

PAOX mediates H_2_O_2_ production during substrate oxidation. Therefore, we analyzed the changes in the intracellular ROS levels in fibroblasts, with or without PAOX expression, using 2′,7′-dichlorofluorescein diacetate (DCF-DA) staining. Because DCF-DA staining shows green fluorescence, a virus in which GFP was deleted was used for the vector control and PAOX expression. We observed that the PAOX expression in the fibroblasts increased the MMP-1 secretion compared with that of the vector control. In the cells stained with DCF-DA, the PAOX expression significantly upregulated the ROS levels compared with that of the vector control (Figure 7A).

The effects of the H_2_O_2_ treatment on the MMP-1 secretion and ROS generation in the fibroblasts were analyzed. The treatment with H_2_O_2_ up to 40 μM increased the MMP-1 secretion, whereas the H_2_O_2_ treatment at 80 μM or higher decreased the same. In addition, the secretion of type I collagen was reduced by H_2_O_2_ concentrations of 80 μM or higher. Increasing the H_2_O_2_ concentrations gradually increased the ROS levels in the fibroblasts. The ROS levels after PAOX expression in the fibroblasts were similar to those after treatment with 40 μM H_2_O_2_. As cytotoxicity was observed at 80 μM H_2_O_2_ or higher, we hypothesized that the decrease in the MMP-1 and type I collagen secretion by H_2_O_2_ at 80 μM or higher was due to the cytotoxicity (Figure 7B).

### 2.7. Antioxidants Decrease PAOX-Mediated Rise in ROS Levels and MMP-1 Secretion in Fibroblasts

**Figure 8:**
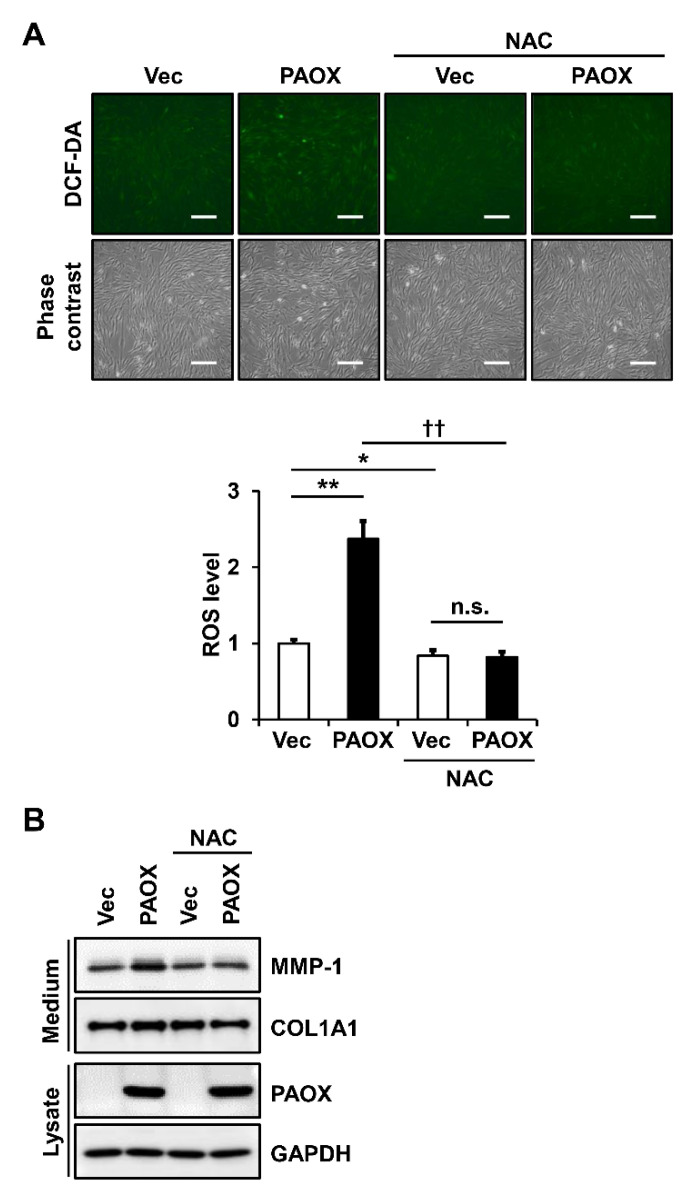
Effect of *N*-acetylcysteine (NAC) treatment on ROS generation and MMP-1 secretion in fibroblasts expressing PAOX. Fibroblasts infected with GFP-deleted vector viruses or GFP-deleted PAOX viruses were incubated in a serum-free and phenol-red-free DMEM for 24 h, with or without 5 mM NAC. (**A**) Intracellular ROS were detected by green fluorescence at 525 nm after staining with 10 μM DCF-DA for 30 min. ROS levels were assessed using ImageJ software, normalized with cell numbers counted using crystal violet staining, and depicted on a graph relative to the ROS levels in untreated vector-infected cells. Each value represents the mean ± standard deviation of three independent experiments. * *p* < 0.05 and ** *p* < 0.01 vs. vector control. †† *p* < 0.01 vs. PAOX. n.s.: not significant. Magnification: ×100. Bar = 200 µm. (**B**) Conditioned media and cell lysates were analyzed using Western blotting with anti-MMP-1, anti-pN-COL1A1, anti-PAOX, and anti-GAPDH antibodies.

To confirm that the increased ROS levels in fibroblasts expressing PAOX are a major cause of increased MMP-1 secretion, we analyzed the MMP-1 secretion in the fibroblasts, with or without PAOX expression, in the presence of an antioxidant. We used *N*-acetylcysteine (NAC), which is an antioxidant that is widely used to protect cells from oxidative stress [19]. As hypothesized, the treatment with 5 mM NAC reduced the PAOX-mediated increase in the ROS levels (Figure 8A) and MMP-1 secretion (Figure 8B), compared with that of the control vector. Therefore, we confirmed that the antioxidant NAC reversed the PAOX-induced increase in the MMP-1 secretion.

### 2.8. PAOX Expression Enhances Activation of AP-1 and NF-κB Signaling Pathways

**Figure 9:**
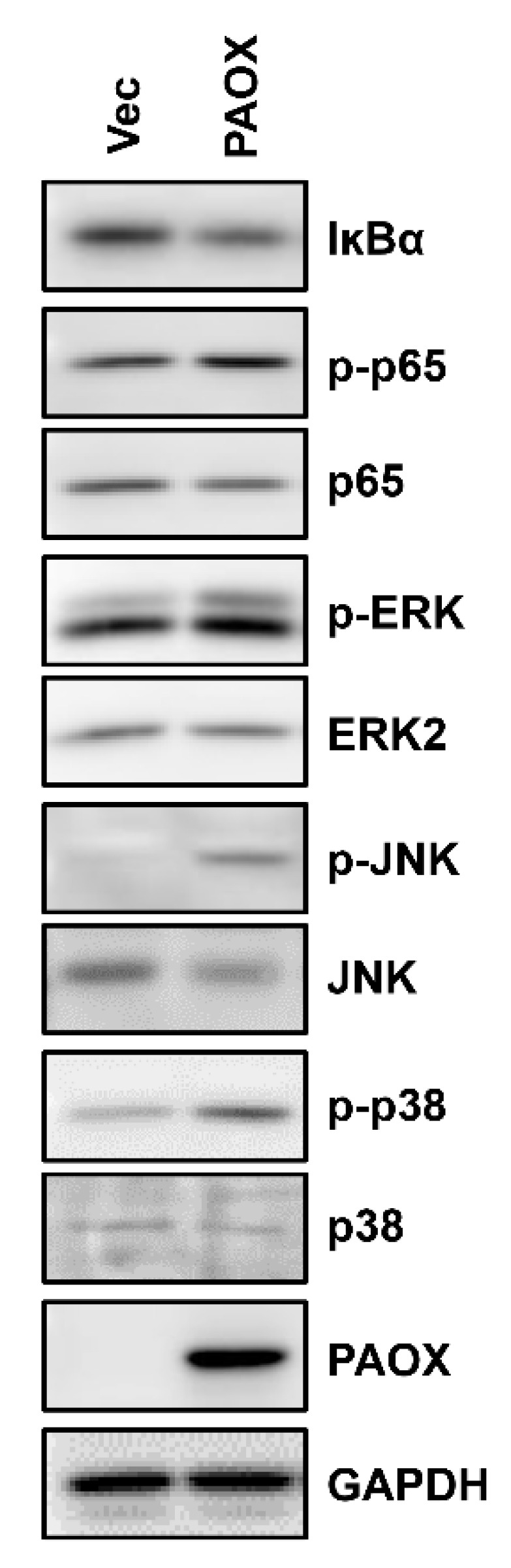
Effect of PAOX expression on phosphorylation of signaling proteins involved in activator protein-1 (AP-1) and nuclear factor kappa-light-chain-enhancer of activated B cells (NF-κB) pathways in fibroblasts. Fibroblasts infected with vector or PAOX viruses were incubated in a serum-free DMEM for 6 h. The protein levels of IκBα and the phosphorylation levels of extracellular signal-regulated kinase (ERK), *c*-Jun *N*-terminal kinase (JNK), p38 mitogen-activated protein kinase (MAPK), and NF-κB were assessed using Western blot analysis.

The transactivation of *MMP-1* is mainly regulated by the transcription factors activator protein-1 (AP-1) and nuclear factor kappa-light-chain-enhancer of activated B cells (NF-κB) [20,21,22,23]. To investigate the signaling pathways involved in *MMP-1* induction by PAOX expression, we analyzed the activation of the signaling proteins involved in the activation of the AP-1 and NF-κB transcription factors in the fibroblasts, with or without PAOX expression. The PAOX expression indicated the increased phosphorylation of extracellular signal-regulated kinase (ERK), *c*-Jun *N*-terminal kinase (JNK), p38 mitogen-activated protein kinase (MAPK), and NF-κB p65, and it decreased the NF-κB inhibitor alpha (IκBα) levels, leading to the activation of AP-1 and NF-κB (Figure 9).

## 3. Discussion

PAOX, an enzyme in the polyamine catabolism pathway, reduces the polyamine levels and generates H_2_O_2_. As the polyamine levels contribute to anti-inflammatory activity, a reduction in the polyamine levels is associated with inflammation. In addition, ROS, including H_2_O_2_, are important signaling molecules involved in inflammatory processes [24,25,26]. Inflammation deteriorates collagen and elastin in the dermis and impairs the barrier function of the skin, leading to skin aging. Therefore, in this study, we investigated the effects of PAOX expression in fibroblasts on ECM modulation and skin aging. We found that PAOX increased the MMP-1 secretion in a 2D culture of fibroblasts. In the 2D-culture system, proMMP-1 is secreted from fibroblasts into the culture medium, but it is poorly processed into mature MMP-1 [27,28]. Thus, secreted proMMP-1 cannot cleave type I collagen, or can only poorly perform. We previously demonstrated that secreted MMP-1 cleaves type I collagen in fibroblasts cultured in a 3D collagen matrix that mimics the dermis [28,29]. Using the 3D-culture system, we observed that the PAOX expression in the fibroblasts increased the cleavage of type I collagen, which was detected by staining with an antibody that recognizes the type I collagen cleavage site. The treatment of TIMP-1 in the 3D culture of fibroblasts blocked the PAOX-induced degradation of type I collagen. Considering that TIMP-1 specifically inhibits various MMPs, including MMP-1 [30], we confirmed that PAOX expression induces MMP-1 expression and increases the degradation of type I collagen in fibroblasts by MMPs, including MMP-1. Furthermore, we observed that the PAOX expression in the fibroblasts increased the *MMP-1* mRNA levels using conventional and real-time RT-PCR analyses, which indicated the *MMP-1* induction at the transcriptional level.

Polyamines have many beneficial effects, such as facilitating an extended lifespan, and cardioprotective and neuroprotective effects. The catabolism of polyamines is catalyzed by enzymes such as PAOX, SSAT, and SMOX, leading to an increase in the SPD:SPM and PUT:SPD ratios, which eventually results in PUT accumulation.

Because PAOX uses acetyl polyamines such as *N*-acetyl SPM and *N*-acetyl SPD as substrates, which are produced by SSAT from SPM and SPD, it induces polyamine catabolism together with SSAT. When we measured the intracellular polyamine levels in the fibroblasts using GC-MS, the PAOX expression drastically increased the PUT levels and significantly increased the SPD levels, while drastically decreasing the SPM levels. Consequently, we found that the increased expression of PAOX alone could induce polyamine catabolism. PUT accumulation in transgenic mice that overexpresses SSAT [31,32], and PUT accumulation with increased SSAT expression in a renal-ischemia/reperfusion-injury mouse model [33], suggest that the induction or activation of PAOX enhances the SSAT/PAOX-mediated polyamine catabolic pathway. Similarly, we found that the PAOX expression in the fibroblasts increased the *SSAT* levels and decreased the *SMOX* mRNA levels. These results indicate that PAOX expression enhances the SSAT/PAOX-mediated polyamine catabolic pathway by inducing SSAT, while decreasing the SMOX-mediated SPM catabolic reaction.

Because PAOX expression in fibroblasts induces changes in the polyamine distribution through polyamine catabolism, we investigated whether altering the polyamine distribution affects the secretion of MMP-1 and type I collagen in fibroblasts. No significant effect in the secretion of MMP-1 and type I collagen was observed by the polyamine treatment in the concentration range that we tested (up to 1 mM), except for a weak decrease (8.9%) in the MMP-1 secretion at 0.1 mM SPM, and a slight increase (5.8%) in the MMP-1 secretion at 1 mM SPM. Among the polyamines, because SPM is known to have the strongest anti-inflammatory effect on cells [34,35,36], a decrease in the MMP-1 secretion at 0.1 mM SPM is speculated to be due to the anti-inflammatory effect of SPM. Nevertheless, the cause of the increase in the MMP-1 secretion at 1 mM SPM remains unknown. Serum amine oxidase in fetal bovine serum (FBS) oxidizes exogenous polyamines (particularly SPD and SPM) to produce cytotoxic products, such as H_2_O_2_, ammonia, and aldehydes [12,37]. Therefore, the increase in the MMP-1 secretion induced by 1 mM SPM could be associated with FBS-derived residual amine oxidase activity. Although a slight change in the MMP-1 secretion by the SPM treatment was observed, the polyamine treatment up to 1 mM did not induce as much of a change in the MMP-1 secretion as that in the PAOX expression.

Because polyamine oxidation by PAOX generates H_2_O_2_, the ROS levels in the fibroblasts were measured using DCF-DA analysis. As hypothesized, the PAOX expression significantly increased the ROS levels in the fibroblasts. The ROS levels induced by the PAOX expression and the exogenous treatment of 40 μM H_2_O_2_ in the fibroblasts were similar. We recently demonstrated that an increase in the ROS levels enhances the MMP-1 expression and type I collagen degradation in dermal fibroblasts [28,29]. Exogenous H_2_O_2_ treatment induces the expressions of MMP-1 and MMP-3 in human retinal pigment epithelium (RPE) cells [38]. These results suggest that an increase in the ROS levels induces MMP-1 expression in fibroblasts and other cell types. Furthermore, we observed that the antioxidant NAC decreased the MMP-1 secretion and ROS levels, which were increased by PAOX. From these results, we found that the H_2_O_2_ produced by PAOX was the main cause of the increased MMP-1 expression in the fibroblasts.

H_2_O_2_ promotes the phosphorylation of ERK1/2, JNK1/2, and p38 MAPK, which consequently activates AP-1 through the phosphorylation and induction of *c*-Fos and *c*-Jun [39]. In addition, H_2_O_2_ activates through the serine phosphorylation of IKKα and IKKβ [40,41], and the Syk-mediated tyrosine phosphorylation of IκBα [42]. Notably, AP-1 and NF-κB play important roles in MMP-1 induction [29]. Similarly, the knockdown or inhibition of the AP-1 and NF-κB transcription factors attenuates MMP-1 expression [21]. We found that the PAOX expression in the fibroblasts increased the phosphorylation of ERK, JNK, p38 MAPK, and NF-κB p65, and the degradation of IκBα. These results strongly suggest that PAOX expression induces MMP-1 expression mainly through the ROS-induced activation of AP-1 and NF-κB.

We demonstrated that the PAOX expression enhanced the SSAT/PAOX-mediated polyamine catabolism by inducing SSAT. Moreover, SSAT is induced by inflammation as well as polyamines [43]. Tumor necrosis factor-α increases both the expression and activation of SSAT via NF-κB activation in non-small cell lung cancer cells [44]. In addition, the SSAT promoter contains NF-κB and AP-1 binding sites [45,46]. Because PAOX activates NF-κB and AP-1, we suggest that PAOX induces SSAT, at least by activating NF-κB and AP-1.

Menopause in women is associated with estrogen depletion, cellular atrophy, degenerative changes in the connective tissue, and a decreased capacity for tissue repair. In particular, decreases in the collagen levels, elasticity, and thickness of the skin were reported in postmenopausal women [47]. The PAOX levels were increased by estrogen receptor 1 (ESR1) loss-of-function in cardiomyocyte-specific ESR1 knockout mice [48]. Furthermore, we recently demonstrated that estrogen suppresses PAOX expression in an ESR2-dependent manner in MCF-7 breast cancer cells [49]. Considering these reports, the lack of estrogen, for example, during menopause, could increase the PAOX levels in the various cell types of the skin. PAOX upregulation, in turn, increases H_2_O_2_ and ROS production and ECM degradation by inducing MMP-1. Although blocking PAOX can be substituted with ROS scavengers or MMP-1 inhibitors, the inhibition or suppression of PAOX might be more effective, as it can maintain the levels of polyamines, which have anti-inflammatory effects. Therefore, we propose that PAOX inhibition or downregulation could be a useful therapeutic strategy against skin aging due to inflammation or menopause.

## 4. Materials and Methods

### 4.1. Antibodies and Reagents

Anti-PAOX antibodies were purchased from MyBioSource (MBS3211539, San Diego, CA, USA), and anti-MMP-1 and pro-collagen α1(I) N-propeptide (pN-ColIα1) antibodies were gifted by Dr. Chung J. H. (Seoul National University College of Medicine, Republic of Korea) [50]. Anti-collagen type I cleavage-site antibodies were purchased from ImmunoGlobe (Himmelstadt, Germany). Anti-IκBα, anti-phospho-NF-κB p65, anti-phospho-JNK, anti-JNK, and anti-phospho-p38 MAPK antibodies were purchased from Cell Signaling Technology (Danvers, MA, USA). Anti-NF-κB p65, anti-p38 MAPK, anti-phospho-ERK, and anti-ERK2 antibodies were purchased from Santa Cruz Biotechnology (Dallas, TX, USA). Anti-glyceraldehyde-3-phosphate dehydrogenase (GAPDH) antibody was purchased from AbClone (Seoul, Korea). Horseradish peroxidase-conjugated goat anti-mouse IgG and goat anti-rabbit IgG were purchased from KOMA Biotech (Seoul, Korea). Rhodamine-conjugated Red-X goat anti-rabbit IgG (H + L) was purchased from Thermo Fisher Scientific (Waltham, MA, USA). Hexadimethrine bromide (polybrene), DCF-DA, and NAC were purchased from Sigma-Aldrich (St. Louis, MO, USA). H_2_O_2_ was purchased from Junsei (Tokyo, Japan). Three polyamines and two internal standards, 1,6-diaminohexane for PUT and d8-SPD for SPD and SPM, were purchased from Sigma-Aldrich. All references and internal standards were prepared at concentrations of 1 mg/mL in 0.1 M HCl, were diluted with 0.1 M HCl, and were stored at -20 °C until use. All organic solvents, which were of analytical and high-performance liquid chromatography grade, were purchased from Burdick & Jackson (Muskegon, MI, USA). TIMP-1 purified from TIMP-1-overexpressing human embryonic kidney 293 (HEK 293) cells was prepared as previously described [18].

### 4.2. Cell Culture

Normal human primary foreskin fibroblasts were purchased from Welgene Inc. (Gyeongsan, Gyeongsangbuk, Korea). Foreskin fibroblasts were grown in Dulbecco’s modified Eagle’s medium (DMEM) (Hyclone, South Logan, UT, USA) supplemented with 10% FBS (Gibco/Thermo Fisher Scientific, Waltham, MA, USA), and HEK 293-T cells were grown in DMEM supplemented with 10% bovine serum (BS) (Gibco/Thermo Fisher Scientific), 100 U/mL of penicillin, and 100 μg/mL streptomycin. The cells were grown at 37 °C in 5% CO_2_ and 95% air.

### 4.3. Construction of Lentiviral Transfer Vectors Harboring Human PAOX cDNA

To construct the pcDNA3.1-FLAG-PAOX vector, a DNA fragment containing the human PAOX coding sequence with an N-terminal FLAG-tag was PCR-amplified using the pCMV-SPORT6-PAOX vector (Miaolingbio, Wuhan, China) as a template and PrimeSTAR GXL DNA polymerase (TaKaRa, Kusatsu, Shiga, Japan). The forward primer, 5′-*ACTTAAGCTTGGTAC*CGCGCG**ATGGACTACAAGGATGACGATGACAAG**GAGTCGACCGGCAGCGTCG-3′, contained 15 nucleotides of pcDNA3.1 vector (italicized), Acc65I site (underlined), Kozak consensus sequence, start codon (bold), FLAG-tag (bold underlined), and 19 nucleotides of PAOX cDNA sequence (NM_152911.4; nt. 59–77). The reverse primer, 5′-*TAGACTCGAGCGGCC*GC**TA**GAGCCTGGGCCTGGGC-3′, contained 15 nucleotides of pcDNA3.1 vector (italicized), NotI site (underlined), and 20 nucleotides of PAOX cDNA sequence (NM_152911.4; nt. 1592–1573), including a stop codon (bold). The PCR product was ligated into the Acc65I and NotI sites of the pcDNA3.1 vector using the In-fusion HD cloning kit (TaKaRa). The subcloning of the FLAG-PAOX coding sequence from pcDNA3.1-FLAG-PAOX into the pHRST-IRES-eGFP vector to generate pHRST-FLAG-PAOX-IRES-eGFP was performed in the same manner as previously described [51].

To construct the pHRST-IRES-ΔeGFP and pHRST-FLAG-PAOX-IRES-ΔeGFP vectors, the eGFP coding sequence was deleted from pHRST-IRES-eGFP and pHRST-FLAG-PAOX-IRES-eGFP, respectively, by DpnI-mediated site-directed mutagenesis using the following primer pair: The forward primer, 5′-GCCACAACCACTAGTGACTTACAAGGCAGCTGTAGATCTTAGC-3′, contained a pHRST-IRES-eGFP vector (DNASU plasmid repository, Tempe, AZ, USA) nt. 3435–3443, SpeI site (underlined), and pHRST-IRES-eGFP vector nt. 4194–4221. The reverse primer, 5′-CCTTGTAAGTCACTAGTGGTTGTGGCCATATTATCATCGT-3′, contained pHRST-IRES-eGFP vector nt. 4204–4194, SpeI site (underlined), and pHRST-IRES-eGFP vector nt. 3443–3421. The constructed plasmids were sequenced to confirm the absence of PCR errors.

### 4.4. Production and Infection of Lentiviruses

HEK293T cells were cotransfected with one of the transfer vectors, pHRST-IRES-eGFP, pHRST-FLAG-PAOX-IRES-eGFP, pHRST-IRES-ΔeGFP, or pHRST-FLAG-PAOX-IRES-ΔeGFP, together with the packaging vector psPAX2, and the envelope vector pMD2.G (Addgene, Cambridge, MA, USA), for 16 h at a transfer vector:packaging vector:envelope vector ratio of 6:3:2. The medium was replaced with fresh medium, and the cells were harvested after 24 h of incubation. The conditioned medium harboring lentiviruses was stored at −70 °C. Fibroblasts were infected with lentiviruses at a virus-to-fresh medium ratio of 2:1 in the presence of 8 µg/mL polybrene for 16 h.

### 4.5. Preparation of Conditioned Media and Cell Lysates for Western Blot Analysis

Fibroblasts were incubated in a serum-free medium for 24 h, and the conditioned media were collected by centrifugation at 2000× *g* for 5 min. Cell pellets were lysed with 1× sodium dodecyl sulfate (SDS) sample buffer to detect PAOX and GAPDH, or with radio-immunoprecipitation assay lysis buffer (50 mM Tris-HCl, pH 7.4, 150 mM NaCl, 1% NP-40, 0.5% sodium deoxycholate, 0.1% SDS) containing 1 mM NaF and 1 mM Na_3_VO_4_ to detect signaling proteins. The conditioned media and cell lysates were adjusted to 1× SDS sample buffer by adding 5× SDS sample buffer. Protein samples were boiled in the presence of 100 mM β-mercaptoethanol and resolved using SDS-polyacrylamide gel electrophoresis (PAGE). Proteins in the gel were blotted onto polyvinylidene fluoride membranes (Millipore, Billerica, MA, USA). The blots were then incubated with primary and secondary antibodies. Immunoreactive signals were detected using Immobilon Western Chemiluminescent HRP Substrate (Millipore) and Amersham ImageQuant 800 (Cytiva, Marlborough, MA, USA).

### 4.6. Analysis of Cleavage and Synthesis of Type I Collagen in a 3D-Culture System

The 3D culture of fibroblasts in the collagen matrix was performed as previously described [29]. Fibroblasts in the 3D culture were fixed with 3.7% paraformaldehyde for 30 min, and were permeabilized in 0.2% Triton-X 100 for 10 min. The cells were stained with Hoechst 33258 (2 μg/mL) for 30 min, blocked with 3% bovine serum albumin for 30 min, immunostained overnight at 4 °C with rabbit anti-type I collagen cleavage-site antibody (2.5 μg/mL), and incubated for 2 h with anti-rabbit IgG (H + L) Rhodamine Red-X (2 μg/mL). Images were obtained using a confocal microscope (LSM880; Zeiss, Oberkochen, Germany) with a 10× Plan-Apochromat objective lens and the Zen software (Zeiss). The excitation wavelengths were 405 nm for Hoechst 33258, 555 nm for Rhodamine Red-X, and 488 nm for GFP. To avoid bias during image acquisition, images were obtained from randomly chosen fields using the same parameters, including the exposure time, laser power, and offset settings. Fluorescence intensity was measured using the ImageJ software (National Institutes of Health, Bethesda, MD, USA).

### 4.7. RNA Isolation and Reverse Transcription Polymerase Chain Reaction (RT-PCR)

Total RNA isolation, cDNA synthesis, and RT-PCR analysis were performed as previously described, with minor modifications [29,52]. Total RNA was isolated from foreskin fibroblasts using TRIZOL (Invitrogen, Carlsbad, CA, USA). Subsequently, cDNA was synthesized from total RNA using oligo (dT)_15_ primers and AMV RTase (Promega, Madison, WI, USA). Conventional PCR amplification was performed in a final volume of 10 μL consisting of 1 pM of the 5′-primer and 3′-primer of each reaction, 0.2 mM dNTPs, 1× Taq PCR buffer, 50 U/mL of Taq polymerase, and cDNAs synthesized from 0.1 µg total RNA. Subsequently, PCR was performed for 25–30 cycles under the following conditions: denaturation at 94 °C for 30 s, annealing at an annealing temperature (Table S1) for 30 s, and extension at 72 °C for 30 s. The PCR products were detected using 5% PAGE analysis and were visualized using ethidium bromide staining. The expression levels of the target genes were normalized to those of GAPDH in the corresponding samples. Real-time PCR was performed at the same annealing temperature as that of conventional PCR using a QuantiTect SYBR Green PCR kit (Qiagen, Hilden, Germany) and the QuantStudio 3 Real-Time PCR system (Applied Biosystems, Foster City, CA, USA).

### 4.8. Determination of Intracellular Polyamine Levels

Intracellular polyamine levels in foreskin fibroblasts were measured using GC-MS. The cells were scraped in ice-cold phosphate-buffered saline (PBS) and sonicated. The cell lysates were rapidly frozen in liquid nitrogen and stored at −70 °C. Sample preparation and quantitative GC-MS analysis were performed as previously described [53].

### 4.9. Measurement of Cellular ROS Levels and Cell Viability

To measure the cellular ROS levels, foreskin fibroblasts were washed twice with PBS and treated with 10 μM of DCF-DA in serum-free DMEM in the dark at 37 °C and 5% CO_2_ in an incubator for 30 min. The cells were then washed twice with PBS and once with phenol-red-free DMEM (Sigma-Aldrich), and they were analyzed using a fluorescence microscope. The fluorescence intensity was measured using ImageJ software. To measure the cell viability, fibroblasts were fixed with 3.7% paraformaldehyde in PBS and stained with 0.005% crystal violet. The stained cells were lysed with 1% SDS, and the absorbance was measured at 600 nm [29].

### 4.10. Statistical Analyses

All data are presented as mean ± standard deviation of at least three independent experiments. Statistical significance was analyzed using an unpaired two-tailed Student’s *t*-test. A *p*-value < 0.05 was considered statistically significant.

## 5. Conclusions

In this study, we found that PAOX expression induced an increase in MMP-1 expression and the degradation of type I collagen in foreskin fibroblasts. PAOX expression changed the intracellular polyamine distribution to decrease SPM and increase PUT, but the exogenous polyamine treatment did not affect the MMP-1 secretion to the same extent as the PAOX expression. Moreover, PAOX expression increased the ROS production via H_2_O_2_ production during polyamine catabolism. The antioxidant NAC reversed the PAOX-mediated ROS production and MMP-1 secretion, which suggests that ROS production is a major contributor to MMP-1 secretion. Additionally, PAOX expression induced AP-1 and NF-κB activation, resulting in *MMP-1* transactivation. Consequently, we propose that the inhibition or suppression of PAOX may help maintain the ECM integrity.

## Data Availability

Data are contained within the article.

## Supplementary Materials

The following supporting information can be downloaded at: https://www.mdpi.com/article/10.3390/ijms231810487/s1.
