# Supplementary material for: Expression of Polyamine Oxidase in Fibroblasts Induces MMP-1 and Decreases the Integrity of Extracellular Matrix
Source: Int J Mol Sci. 2022 Sep 10;23(18):10487. doi: 10.3390/ijms231810487 (PMC9504367; doi:10.3390/ijms231810487)
Supplement: Supplementary file 1 [file ijms-23-10487-s001.zip › ijms-1894526-supplementary.pdf]

Supplementary materials

Expression of Polyamine Oxidase in Fibroblasts Induces MMP-1 and Decreases the Integrity of Extracellular Matrix

Hae Dong Jeong [1], Jin Hyung Kim [1], Go Eun Kwon [2], and Seung-Taek Lee [1,*]

[1] Department of Biochemistry, College of Life Science and Biotechnology, Yonsei University, Seoul 03722, Korea

[2] Molecular Recognition Research Center, Korea Institute of Science and Technology, Seoul 02792, Korea

* Correspondence: stlee@yonsei.ac.kr; Tel.: +82221232703

Table S1. Primer sequences used for reverse transcription-polymerase chain reaction of *MMP-1, COL1A1, PAOX, SMOX, SSAT,* and *GAPDH* mRNAs.

**Gene Symbol**	**Nucleotide Sequence**	**Nucleotide Position**	**Annealing Temp. (°C)**	**GenBank #**
MMP1-F	5´-GTACTGATATAATTTAGTTC-3′	1656–1675	45	NM_002421
MMP1-R	5′-GTTATCCCTTGCCTATCTAG-3′	1908–1889		
COL1A1-F	5′-ACAGCGTCACTGTCGATGGCTG-3′	4341–4372	55	NM_000088
COL1A1-R	5′-GGAGGGAGTTTACAGGAAGCAGACAG-3′	4522–4497		
PAOX-F	5′-AAGAGCGTCCTGCGGTCTCG-3′	1313–1332	60	NM_152911.4
PAOX-R	5′-CGTCCGTCGTGGAGTAAAACGT-3′	1501–1484		
SMOX-F	5′-ATGCAGGTGCTGTTTTCCGGTGA-3′	1710–1732	65	NM_175839.3
SMOX-R	5′-GGTACATCTCAATGAGGCGGGC-3′	1818–1797		
SSAT-F	5′-GCAGCAGCATGCACTTCTTGGTA-3′	544–566	60	NM_002970.4
SSAT-R	5′-AGTCTCCAACCCTCTTCACTGGA-3′	646–624		
GAPDH-F	5′-ACTGCTTAGCACCCCTGGCCA-3′	488–508	55	BC023632
GAPDH-R	5′-TTGGCAGTGGGGACACGGAAG-3′	740–720		

F: forward primer and R: reverse primer
